# Comparison of Argon and Oxygen Plasma Treatments for Ambient Room-Temperature Wafer-Scale Au–Au Bonding Using Ultrathin Au Films

**DOI:** 10.3390/mi10020119

**Published:** 2019-02-13

**Authors:** Michitaka Yamamoto, Takashi Matsumae, Yuichi Kurashima, Hideki Takagi, Tadatomo Suga, Toshihiro Itoh, Eiji Higurashi

**Affiliations:** 1 National Institute of Advanced Industrial Science and Technology (AIST), 1-2-1 Namiki, Tsukuba, Ibaraki 305-8564, Japan; t.matsumae@aist.go.jp (T.M.); y-kurashima@aist.go.jp (Y.K.); takagi.hideki@aist.go.jp (H.T.); 2 Graduate School of Frontier Sciences, The University of Tokyo, 5-1-5 Kashiwanoha, Kashiwa-shi, Chiba 277-8563, Japan; toshihiro-itoh@edu.k.u-tokyo.ac.jp; 3 School of Engineering, The University of Tokyo, 7-3-1 Hongo, Bunkyo-ku, Tokyo 113-8656, Japan; suga@pe.t.u-tokyo.ac.jp

**Keywords:** heterogeneous integration, wafer bonding, low-temperature bonding, Au–Au bonding, ultrathin Au films, Ar plasma treatment, O_2_ plasma treatment

## Abstract

Au–Au surface activated bonding is promising for room-temperature bonding. The use of Ar plasma vs. O_2_ plasma for pretreatment was investigated for room-temperature wafer-scale Au–Au bonding using ultrathin Au films (<50 nm) in ambient air. The main difference between Ar plasma and O_2_ plasma is their surface activation mechanism: physical etching and chemical reaction, respectively. Destructive razor blade testing revealed that the bonding strength of samples obtained using Ar plasma treatment was higher than the strength of bulk Si (surface energy of bulk Si: 2.5 J/m^2^), while that of samples obtained using O_2_ plasma treatment was low (surface energy: 0.1–0.2 J/m^2^). X-ray photoelectron spectroscopy analysis revealed that a gold oxide (Au_2_O_3_) layer readily formed with O_2_ plasma treatment, and this layer impeded Au–Au bonding. Thermal desorption spectroscopy analysis revealed that Au_2_O_3_ thermally desorbed around 110 °C. Annealing of O_2_ plasma-treated samples up to 150 °C before bonding increased the bonding strength from 0.1 to 2.5 J/m^2^ due to Au_2_O_3_ decomposition.

## 1. Introduction

Low-temperature wafer bonding is increasingly required due to the increasing use of heterogeneous integrations [1,2,3]. An effective approach to such bonding is to use intermediate layers [4,5]. Gold (Au) is a good candidate material for these bonding layers because of its high electrical and thermal conductivity, good deformability, and high oxidation resistance.

Several Au–Au bonding techniques have been investigated to achieve low-temperature bonding, including thermocompression bonding [6,7,8,9,10,11,12], atomic diffusion bonding [4,13,14], and surface activated bonding (SAB) [15,16,17,18,19]. With SAB, the surfaces to be bonded are activated by plasma pretreatment and then bonded at low temperature (<150 °C). Furthermore, the use of Au for the bonding layer enables bonding in ambient air because gold oxide (Au_2_O_3_) is the only metal oxide that has a positive formation enthalpy (+19.3 kJ/mol) [20]. The use of SAB with little contact pressure at room temperature in ambient air using ultrathin (<50 nm) Au films with smooth (root mean square (RMS) <0.50 nm) surfaces was recently shown to result in reliable Au–Au bonding [17].

While Ar plasma is commonly used to activate Au surfaces by physically bombarding them with Ar ions or atoms, O_2_ plasma is commonly used to clean surfaces because it can eliminate organic contaminants by chemical reaction [21,22]. It is also used for pretreatment before Au–Au bonding; O_2_ plasma treatment has been reported to more effectively improve Au wire bond interfacial adhesion than Ar plasma treatment [23,24]. However, the Au–Au bonding may be weakened by the mixing of O_2_ gas into the plasma processing gas [25,26]. The effect of plasma treatment, and especially the effect of O_2_ plasma treatment on Au–Au bonding, thus needs further investigation.

This paper reports the investigation of Ar plasma vs. O_2_ plasma as a pretreatment method for room-temperature wafer-scale Au–Au bonding using ultrathin Au films (<50 nm) in ambient air. The main difference between Ar plasma and O_2_ plasma is their surface activation mechanism: physical etching and chemical reaction, respectively. The investigation included an analysis of Au surfaces treated with Ar or O_2_ plasma.

## 2. Materials and Methods

Silicon substrates with Au film and a Ti adhesion layer were used for analysis and bonding. A Ti adhesion layer followed by Au thin film was deposited on a Si substrate or a thermally oxidized Si substrate by direct current (DC) sputtering at a substrate temperature of 25 °C. The Ar or O_2_ plasma used to activate the surfaces was generated by applying 200 W radio frequency (RF) power at 60 Pa. Both plasmas were generated using the same equipment; only the process gas was changed.

The effect of each plasma treatment on the surface properties was determined by evaluating the surface roughness, hydrophilicity, electrical characteristics, and chemical state of the Au surfaces before and after each treatment. Surface roughness was measured using an atomic force microscope (AFM, Hitachi High-Tech Science Co., Tokyo, Japan, L-trace) and Si wafers with Au thin film (thickness: 15 nm) and a Ti adhesion layer (thickness: 5 nm). The AFM measurement was performed with a scan area of 500 nm × 500 nm and a resolution of 256 × 256 pixels. Hydrophilicity was evaluated by measuring the contact angle of the Au surfaces as measured with a contact angle meter (Kyowa Interface Science Co, Ltd., Saitama, Japan, PCA-11). Pure water (1.5 µL) was dropped on the Au surfaces, and the contact angle was calculated using the half-angle method. The electrical characteristics were evaluated by four-point measurement of the sheet resistance using a resistivity processor (NPS Inc., Tokyo, Japan, Model Σ) and Si wafers with Au thin film (thickness: 15 nm) and a Ti adhesion layer (thickness: 5 nm). The effect of the plasma treatment time was measured by irradiating plasma onto the same wafer several times. The surface chemical states were determined using X-ray photoelectron spectroscopy (XPS) analysis and thermal desorption spectrometry (TDS) analysis. The XPS analysis was performed using an X-ray photoelectron spectroscope (JEOL Ltd., Tokyo, Japan, JPS9200-T). A non-monochromatic Mg Kα source (photon energy: 1253.6 eV) was used at an operating power of 10 kV × 10 mA. Smoothing, Shirley-type background subtraction, and charge correction using the Au 4f_7/2_ binding energy were performed. The TDS analysis was performed using a thermal desorption spectrometer (ESCO Ltd., Tokyo, Japan, EMD-WA1000S). Thicker Au films (thickness: 300 nm) with Ti adhesion layers (thickness: 5 nm) deposited on thermally oxidized Si substrates were used to avoid the effect of diffusion from the adhesion layer or Si substrate during annealing. The temperature was increased at a rate of 30 °C/min and was corrected by measurement using a dummy Si substrate.

Four-inch Si wafers with Au thin film (thickness: 15 nm) and a Ti adhesion layer (thickness: 5 nm) were bonded after Ar or O_2_ plasma treatment in ambient air by overlapping two wafers and pinching the wafers together with tweezers. Bond front propagation was observed using an infrared (IR) transmission setup. The effects of plasma treatment time and air exposure time after plasma treatment on self-propagation of the bonding area and on bonding strength were investigated. The effects of annealing before bonding on the self-propagation of the bonding area and on bonding strength were also investigated by annealing wafers that had been plasma-treated up to 150 °C for 10 min in vacuum and then bonding them together in ambient air at room temperature. 

Bonding strength was measured using destructive razor blade testing, also known as the crack opening or double cantilever beam method [27,28]. This testing method is an effective way to evaluate the energy of the bonded surfaces (i.e., surface energy). It is performed by inserting a razor blade between the bonded wafer pairs and observing the crack length along the bonded interface. The surface energy *γ* (J/m^2^) (i.e., half the bonding energy *G*) was calculated under plane strain conditions and the assumptions of beam theory using

(1) $$\gamma =\frac{3}{32}\cdot \frac{ET_{b}{}^{2}T_{w}{}^{3}}{L^{4}},$$

where *E* (Pa) is the Young’s modulus of the wafer, *T_b_* (m) is the blade thickness, *T_w_* (m) is the wafer thickness, and *L* (m) is the crack length [27]. In this experiment, Si substrates with a thickness of 

525 μm and a blade with a thickness of 100 µm were used. For calculation, we assumed that Si is isotropic, with Young’s modulus of elasticity *E* = 169 GPa [29,30]. Although the measurement should be conducted using beam-shaped samples in order to accurately determine the surface energy [31], we used whole bonded wafer pairs because the fabrication of beam-shaped samples is particularly difficult for weakly bonded wafer pairs. The crack length along the bonded interface was observed with an IR camera in a standard clean room atmosphere. Since values obtained from Equation (1) are affected by the crack detection resolution, anisotropic mechanical properties, and humidity environment [28,30], the comparison of absolute energy values with published results requires careful consideration.

## 3. Results

### 3.1. Surface Analysis

#### 3.1.1. AFM Measurement

**Figure 1:**
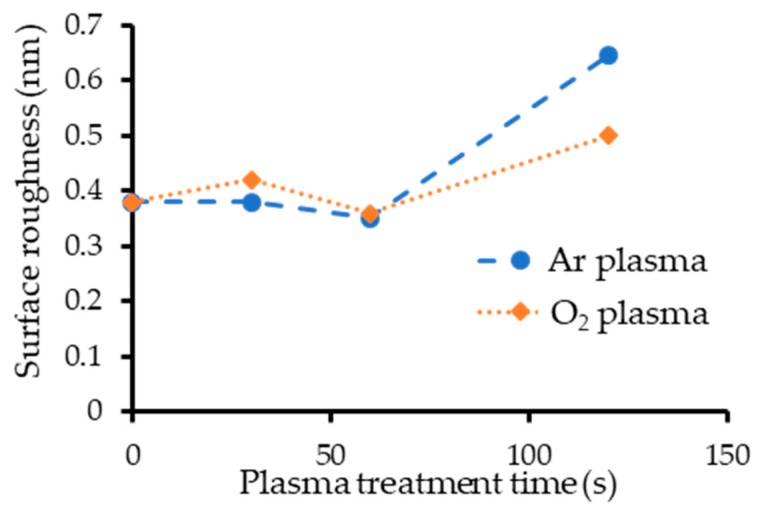
Root mean square (RMS) surface roughness vs. plasma treatment time.

The measured RMS surface roughness for different plasma treatment times (30, 60, 120 s) are plotted in Figure 1. The roughness before plasma treatment was 0.38 nm. Both the Ar and O_2_ plasma treatments of 60 s or less had little effect on surface roughness, while both treatments of 120 s increased it. This indicates that a short plasma treatment time is important for achieving low-temperature bonding.

#### 3.1.2. Contact Angle Measurement

**Figure 2:**
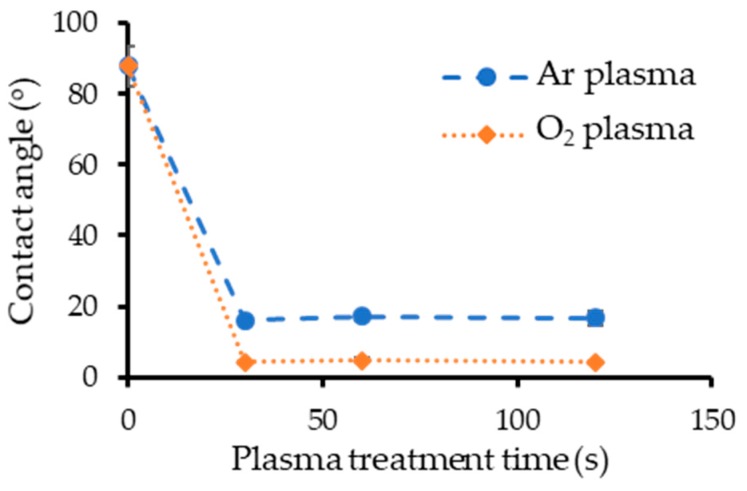
Measured contact angle vs. plasma treatment time.

**Figure 3:**
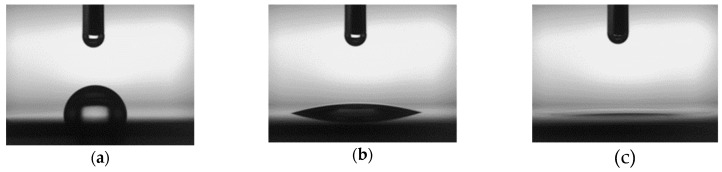
Typical images of measured contact angle: (**a**) without plasma treatment; (**b**) with 30 s Ar plasma treatment; (**c**) with 30 s O_2_ plasma treatment.

The measured contact angles for different plasma treatment times (30, 60, 120 s) are shown in Figure 2. The Au films had been exposed to air for over 1 month, and the measurement was performed immediately after plasma treatment. Typical images of the measured contact angle are shown in Figure 3. Figure 3b,c was taken 30 s after plasma treatment. Before treatment, the contact angle was about 80–100°, and the Au surfaces were hydrophobic. Both treatments reduced the contact angle, which means that the Au surfaces became hydrophilic with plasma treatment. The contact angle for Au surfaces with O_2_ plasma treatment was less than 5°, which is smaller than that with Ar plasma treatment. The treatment times were the same.

**Figure 4:**
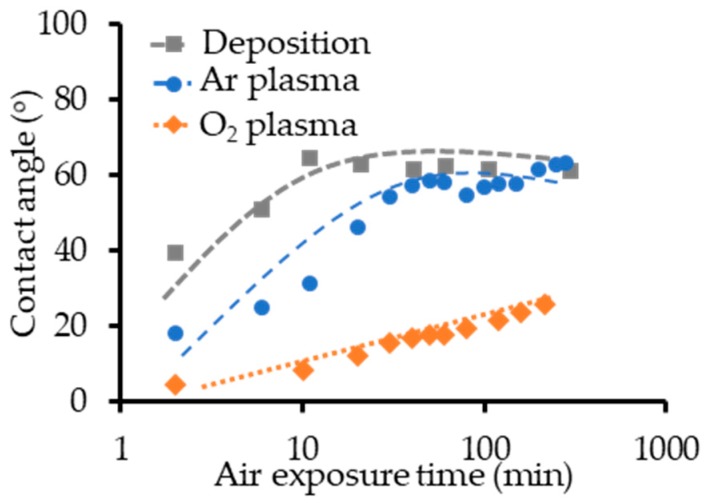
Changes in contact angle after exposure in air following Au film deposition and plasma treatment.

The changes in the contact angle with the air exposure time after Au film deposition and after plasma treatment are plotted in Figure 4. Previous studies reported that Au film is hydrophilic immediately after deposition and that the Au surface quickly becomes hydrophobic after exposure in air [32,33]. The measured change in the contact angle after deposition agrees well with the results of previous studies. The contact angles of Au surfaces that received Ar plasma treatment increased rapidly after exposure in air, similar to the results for the Au film immediately after deposition. In contrast, the rate of increase was much lower for Au surfaces that received O_2_ plasma treatment.

#### 3.1.3. Sheet Resistance Measurement

**Figure 5:**
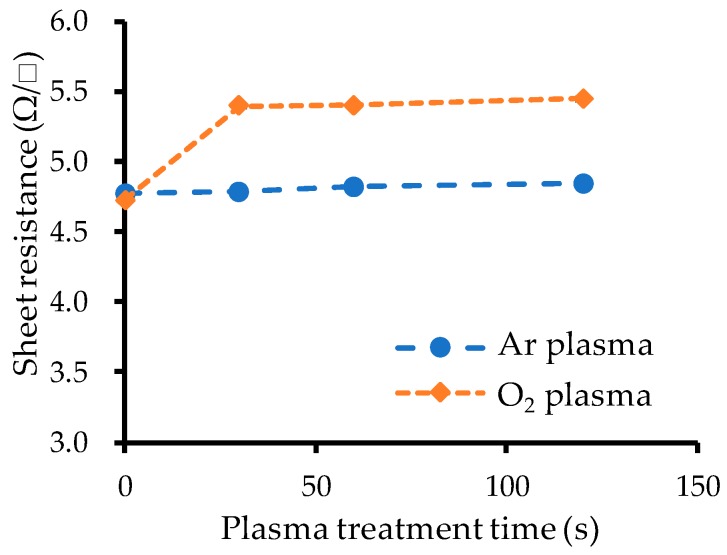
Measured sheet resistance vs. plasma treatment time.

The relationship between the measured sheet resistance and the plasma treatment time is plotted in Figure 5. Before plasma treatment, the sheet resistance was about 4.7 Ω. While Ar plasma treatment did not change the resistance, 30 s O_2_ plasma treatment increased it to about 5.4 Ω. Additional O_2_ plasma treatment time did not increase it.

#### 3.1.4. XPS Analysis

**Figure 6:**
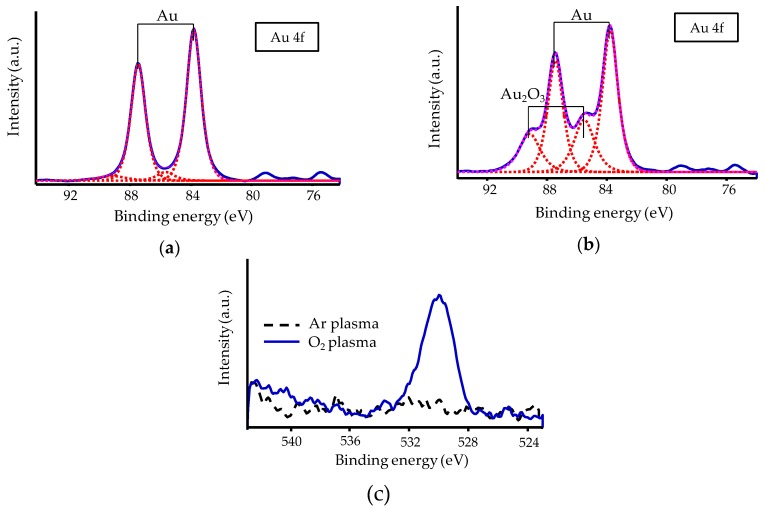
X-ray photoelectron spectroscopy (XPS) spectra of Au surfaces that received different plasma treatments: (**a**) relative peak intensity in the Au 4f region of surface that received Ar plasma treatment; (**b**) relative peak intensity in the Au 4f region of surface that received O_2_ plasma treatment; (**c**) relative peak intensities in the O 1s region of surface that received Ar and O_2_ plasma treatments.

The Au surface conditions were evaluated by using XPS analysis to investigate Au surfaces after Ar or O_2_ plasma treatment. The treatment time was set to 60 s. The relative peak intensities in the Au 4f region are plotted in Figure 6a,b, and the relative peak intensities in the O 1s region are plotted in Figure 6c. The results plotted in (a) show that the Au surfaces that received Ar plasma treatment were pure Au, while those plotted in (b) show that an Au_2_O_3_ peak appeared for the Au surfaces that received O_2_ plasma treatment. The results plotted in (c) show that the O_2_ plasma treatment increased the oxygen contamination intensity.

#### 3.1.5. TDS Analysis

**Figure 7:**
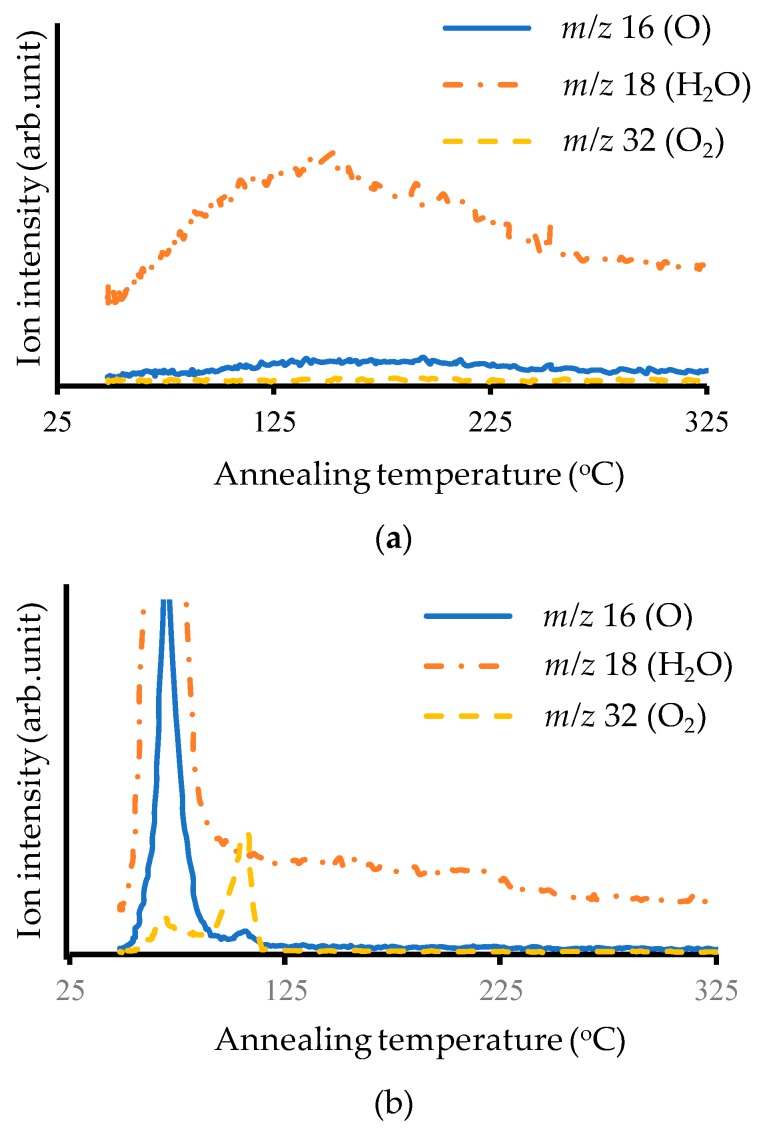
Results of TDS analysis: (**a**) with Ar plasma treatment; (**b**) with O_2_ plasma treatment.

The results of the TDS analysis are plotted in Figure 7. There was little desorption from the Au surface with the Ar plasma treatment. In contrast, with the O_2_ plasma treatment, mass-to-charge ratio (*m*/*z*) peaks of 16 and 18 were detected at annealing temperatures around 70 °C, and *m*/*z* peaks of 16 and 32 were detected at annealing temperatures around 110 °C. Since *m*/*z* 16, 18, and 32 correspond to O, H_2_O, and O_2_, it appears that H_2_O was desorbed around 70 °C from the Au surfaces that received O_2_ plasma treatment, while O_2_ was desorbed around 110 °C.

### 3.2. Bonding Results

#### 3.2.1. Effect of Plasma Treatment Time

**Figure 8:**
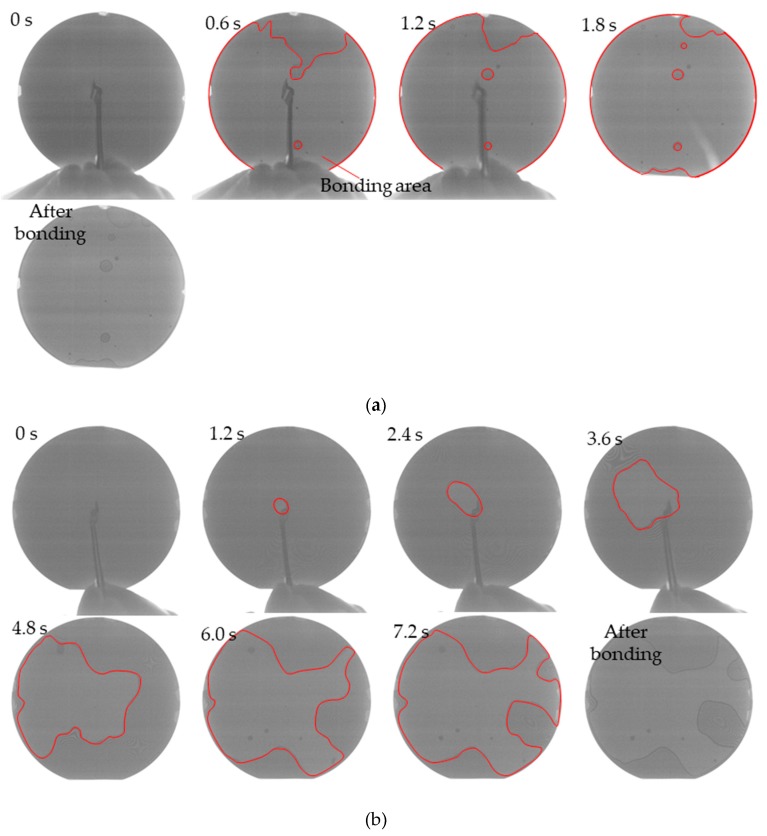
Images of increasing bonding area: (**a**) with 60 s Ar plasma treatment; (**b**) with 60 s O_2_ plasma treatment.

To determine the effect of the plasma treatment time, Au–Au bonding with or without plasma treatment was performed. The treatment times were 30 s or 60 s because a longer treatment time increased the surface roughness. Bonding was performed immediately after plasma treatment. Without plasma treatment, bonding front propagation did not occur, and bonding did not succeed. With plasma treatment, bonding front propagation occurred, and bonding succeeded. IR images of typical bond front propagation between two Si wafers for a plasma treatment time of 60 s are shown in in Figure 8. The contrast and brightness of the images were adjusted, and the edges of the bonding area were marked to make it more visible. With Ar plasma treatment, the bonding area quickly expanded across the entire 4-inch wafer after pushing the center. With O_2_ plasma treatment, the bonding area expanded much more slowly.

The results of the destructive razor blade testing are summarized in Table 1. With Ar plasma treatment (30 s and 60 s), the bonding was sufficient to break the substrate, meaning that strong bonding was achieved. With O_2_ plasma treatment, although much of the area was bonded, the bonding strength (surface energy) was only about 0.1–0.2 J/m^2^, lower than the surface energy of Au (1.6 J/m^2^) [34]. Debonding thus occurred between the Au–Au bonding interfaces.

**Figure 9:**
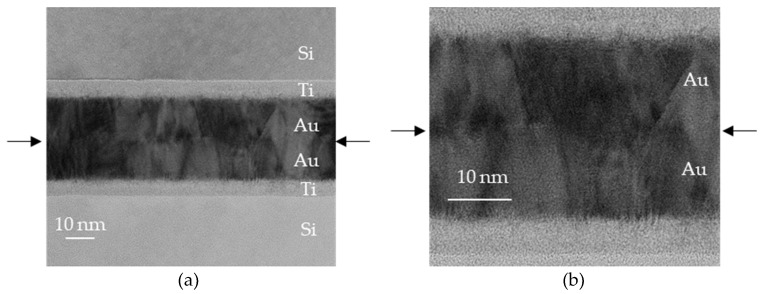
Transmission electron microscope (TEM) images of Au–Au bonding interface after 30 s Ar plasma treatment. Arrows indicate original interface: (**a**) low magnification; (**b**) high magnification.

Transmission electron microscope (TEM) images of a bonded sample that received Ar plasma treatment are shown in Figure 9. Bonding was achieved at the atomic level, and part of the grain boundary grew beyond the bonding interface.

#### 3.2.2. Effect of Air Exposure

To investigate the effect of air exposure after plasma treatment, bonding was performed after air exposure. The bonding of Au films immediately after deposition was also compared. Because Au films immediately after deposition have clean and activated surfaces, bonding can be performed at room temperature. This bonding technique is usually called atomic diffusion bonding [4,13,14]. The effect of air exposure time on the occurrence of self-propagation of the bonding area and on the bonding strength of atomic diffusion bonding for Au films immediately after deposition is summarized in Table 2. Self-propagation occurred even after 1 h of air exposure. Though the bonding strength decreased as the exposure time increased, sufficient bonding strength (greater than the surface energy of bulk Si (2.5 J/m^2^) [35]) was obtained even after 1 h of air exposure. 

The effect of air exposure time for surface activated bonding is summarized in Table 3. In the cases where self-propagation did not occur, bonding was achieved by pushing the whole wafer with tweezers. With Ar plasma treatment, self-propagation did not occur after 30 min of air exposure. Moreover, the bonding strength greatly decreased as the air exposure time increased. With O_2_ plasma treatment, while self-propagation occurred even after 1 hour of air exposure, the bonding strength was lower under all conditions.

#### 3.2.3. Room-Temperature Wafer-Scale Au–Au Bonding After Annealing

Analysis of Au surfaces that received O_2_ plasma treatment revealed that Au_2_O_3_ formed and that O_2_ desorbed around 110 °C. This indicates that bonding after annealing at over 110 °C increases the bonding strength of samples that receive O_2_ plasma treatment. Here, annealing up to 150 °C after plasma treatment was investigated: Ar or O_2_ plasma was irradiated onto Au surfaces for 60 s, and then the wafers were annealed at 150 °C for 10 min in vacuum. After annealing, the wafers were bonded in ambient air at room temperature. The entire annealing process took about one hour.

Self-propagation did not occur in either the Ar or O_2_ plasma-treated samples. The wafers were thus bonded by pushing the entire wafers with tweezers. Although the bonding strength of samples that received Ar plasma treatment exceeded the surface energy of bulk Si (2.5 J/m^2^), the annealing reduced the bonding strength, as evidenced by the breaking of the wafers during blade testing for wafers that had not been annealed. The bonding strength of samples that received O_2_ plasma treatment increased substantially (from 0.1 J/m^2^ without annealing to 2.5 J/m^2^ with annealing). To investigate whether Au_2_O_3_ was desorbed, XPS analysis of annealed Au surfaces after O_2_ plasma treatment was also performed. The results did not reveal an Au_2_O_3_ peak, meaning that Au_2_O_3_ formed by O_2_ plasma treatment impedes Au–Au bonding. To investigate the change in surface roughness due to annealing, AFM analysis was also performed. The results did not reveal any changes in surface roughness.

## 4. Discussion

While Ar plasma treatment improved room-temperature wafer-scale Au–Au bonding strength, O_2_ plasma treatment did not. The O_2_ plasma treatment reduced the contact angle to less than 5° and increased the sheet resistance. XPS analysis showed that the O_2_ plasma treatment formed Au_2_O_3_ on the Au surface. This indicates that the changes in contact angle and sheet resistance were both apparently caused by oxidation of the Au surfaces and that the Au_2_O_3_ formed by the O_2_ plasma treatment impeded Au–Au bonding.

Because TDS analysis showed that Au_2_O_3_ desorbed at around 110 °C, bonding after annealing up to 150 °C was performed. While the bonding strength of samples that received Ar plasma treatment was reduced by the annealing, that of samples that received O_2_ plasma treatment was improved from 0.1 to 2.5 J/m^2^. This also demonstrates that the Au_2_O_3_ formed by O_2_ plasma treatment impeded Au–Au bonding. The decrease in bonding strength with Ar plasma treatment may have been due to the annealing processing time (~1 h).

The effects of air exposure after Au film deposition or plasma treatment on the contact angle and bonding strength were also investigated. The changes in surface hydrophilicity due to contact angle measurement of water on Au surfaces that received Ar plasma treatment were similar to those on Au surfaces immediately after deposition. The bonding strength of samples that were exposed in air following Ar plasma treatment decreased more rapidly than that of samples bonded immediately after deposition. Further research on the effects of air exposure and the relationship between surface conditions and bonding strength is necessary.

Previous studies found that O_2_ plasma treatment improved Au–Au bonding strength [23,24], possibly because Au_2_O_3_ desorbed due to the high bonding temperature (150 °C) or ultrasonic vibrations.

## 5. Conclusions

Pretreatment using Ar or O_2_ plasma was investigated for ambient room-temperature wafer-scale Au–Au bonding using ultrathin Au films. Surface activation by Ar plasma is mainly due to physical etching by Ar ions, while surface activation by O_2_ plasma is mainly due to chemical reaction. The O_2_ plasma treatment increased the sheet resistance of the Au surfaces but did not increase the bonding strength. XPS analysis revealed that Au_2_O_3_ formed on the Au surfaces that received O_2_ plasma treatment, and TDS analysis revealed that annealing at over 110 °C is necessary for desorption of Au_2_O_3_. Annealing up to 150 °C before bonding increased the bonding strength of samples that received O_2_ plasma treatment, demonstrating that Au_2_O_3_ impedes strong Au–Au bonding.

On the other hand, Ar plasma treatment increased the Au–Au bonding strength enough for the Si substrate to be broken in destructive razor blade testing. Although an Au surface that receives Ar plasma treatment is more activated than one that receives O_2_ plasma treatment, bonding should be performed immediately after plasma treatment.

## Figures and Tables

**Table 1:** Results of room-temperature wafer-scale Au–Au bonding with Ar or O_2_ plasma treatment.

Types of Plasmas	Plasma Treatment Time (s)		
	0 (Without Plasma)	30	60
Ar plasma	Bonding failed	Wafer broken (High bonding strength)	Wafer broken (High bonding strength)
O_2_ plasma	Bonding failed	0.2 J/m^2^	0.1 J/m^2^

**Table 2:** Effect of air exposure time on the occurrence of self-propagation of the bonding area and on the bonding strength of atomic diffusion bonding for Au films immediately after deposition.

Evaluation Points	Air Exposure Time			
	Within 5 min	10 min	30 min	1 h
Occurrence of self-propagation	Yes	Yes	Yes	Yes
Bonding strength (J/m^2^)	Wafer broken (High bonding strength)	Wafer broken (High bonding strength)	Wafer broken (High bonding strength)	>2.5

**Table 3:** Effect of air exposure time on the occurrence of self-propagation of the bonding area and on the bonding strength of surface activated bonding.

Types of Plasmas	Evaluation Points	Air Exposure Time			
		Within 5 min	10 min	30 min	1 h
Ar plasma	Occurrence of self-propagation	Yes	Yes	No	No
	Bonding strength (J/m^2^)	Wafer broken (High bonding strength)	>2.5	2.0	0.1
O_2_ plasma	Occurrence of self-propagation	Yes	Yes	Yes	Yes
	Bonding strength (J/m^2^)	0.1	0.1	0.1	0.1
